# Comparative Evaluation of SARS-CoV-2 RNA Concentration and Normalization Strategies in Prison Wastewater: Implications for Viral Dynamics in Confined Environments

**DOI:** 10.3390/v18050563

**Published:** 2026-05-15

**Authors:** Raheel Nazakat, Nabilla Athieqa Mahdzar, Amirul Haziq Azwan, Reethiya Letchumanan, Siti Aishah Rashid

**Affiliations:** 1 Environmental Health Research Centre, Institute for Medical Research, National Institutes of Health, Ministry of Health Malaysia, Shah Alam 40170, Malaysia; raheel@moh.gov.my (R.N.); nabillaathieqa@moh.gov.my (N.A.M.); amrlhzq07@gmail.com (A.H.A.); 2 Malaysian Prison Department, Ministry of Home Affairs of Malaysia, Kajang Emenyih Bypass, Kajang 43000, Malaysia; reethiya@prison.gov.my

**Keywords:** wastewater-based epidemiology, SARS-CoV-2, direct capture, electronegative membrane filtration, PMMoV normalization

## Abstract

Background: Wastewater-based epidemiology (WBE) is a valuable population-level surveillance tool for monitoring SARS-CoV-2 circulation. However, evidence on optimal viral concentration approaches in confined institutional settings such as prisons remains limited. This study aimed to compare the performance of Direct Capture (DC) and Electronegative Membrane Filtration (EMF) for SARS-CoV-2 RNA detection in wastewater from a prison facility in Selangor, Malaysia. Methods: Composite wastewater samples collected over 18 weeks (April–August 2023; *n* = 50) were analysed by RT-dPCR targeting the N1 and N2 gene regions, with concentrations normalized to pepper mild mottle virus (PMMoV). DC consistently outperformed EMF across both gene targets. Median concentrations obtained using DC were 11.09 × 10^3^ copies L^−1^ (N1) and 3.43 × 10^3^ copies L^−1^ (N2), compared with 0.70 × 10^3^ copies L^−1^ (N1) and 0.48 × 10^3^ copies L^−1^ (N2) using EMF. Detection frequencies were higher with DC (N1: 94%, N2: 84%) than with EMF (N1: 88%, N2: 76%). Paired statistical analysis confirmed significant differences between methods (N1: *p* = 2.3 × 10^−7^; N2: *p* = 9.4 × 10^−5^), and Bland–Altman analysis demonstrated systematic underestimation by EMF (mean bias −1.15 log_10_ for N1; −0.87 log_10_ for N2), indicating that the methods are not analytically interchangeable. Conclusions: Normalization reduced absolute SARS-CoV-2 RNA concentrations while preserving temporal trends, supporting its use to improve comparability across sampling periods. Overall, these findings demonstrate that DC combined with N1 detection provides a more sensitive and reliable approach for SARS-CoV-2 WBE in confined settings, underscoring the importance of methodological optimization to strengthen early-warning capacity in high-risk environments.

## 1. Introduction

The Coronavirus disease 2019 (COVID-19) pandemic revealed significant global and national vulnerabilities in responding to a rapidly evolving respiratory virus. Malaysia, like many countries, experienced overwhelming hospital bed occupancy, repeated movement control orders, and major institutional outbreaks, including large prison clusters reported by the Ministry of Health throughout 2020–2021 [1,2,3,4]. As the nation transitions into the Living with COVID phase, these events underscore the need for sustained, population-level surveillance systems that operate independently of clinical testing compliance [5].

From an environmental virology perspective, wastewater represents an integrated reservoir of viral material shed in feces, urine, and saliva enabling detection in wastewater prior to symptom onset [6]. Severe acute respiratory syndrome coronavirus 2 (SARS-CoV-2) appears in wastewater early in infection, often before symptoms or clinical reporting, positioning wastewater-based epidemiology (WBE) as a sensitive early-warning tool [7,8]. Studies have shown that WBE can detect both symptomatic and silent transmission, yet most research has focused on municipal systems rather than high-risk confined settings such as prisons, where overcrowding, limited ventilation, and constrained healthcare access amplify transmission risk [9,10]. Consequently, the implementation of WBE in carceral facilities offers a proactive approach to monitor viral dynamics, mitigate outbreaks, and inform public health interventions within these vulnerable populations [11].

During the pandemic, clinical surveillance relied heavily on nasopharyngeal and oropharyngeal swabs or antigen kits. While diagnostically valuable, these approaches are resource-intensive and depend on individuals’ willingness to be tested [12]. These limitations of clinical testing are even more pronounced in carceral facilities, where restricted healthcare access and low routine testing uptake render individual surveillance impractical [13,14]. In contrast, although WBE has proven effective, accurate interpretation of viral dynamics in wastewater relies heavily on the methodological framework applied. Wastewater matrices, laden with organic debris, chemicals, and polymerase chain reaction (PCR) inhibitors, present substantial obstacles to precise viral detection via RT-PCR-based assays. The concentration step is the major bottleneck of successful SARS-CoV-2 genome recovery. Accordingly, a broad array of concentration methods has been assessed, including ultrafiltration, ultracentrifugation, polyethylene glycol precipitation, aluminium flocculation, skimmed-milk flocculation, and electronegative membrane filtration [15,16,17]. In selecting a surveillance protocol, considerations must extend beyond analytical performance to encompass cost, operational simplicity, and the technical capacity of the implementing team.

To address these gaps, this study compares two SARS-CoV-2 RNA concentration methods, Direct Capture (DC) and Electronegative Membrane Filtration (EMF) using digital PCR (dPCR) for accurate quantification. We assess N1 and N2 gene detection between EMF and DC concentration method, normalized and non-normalized viral loads with Pepper mild mottle virus (PMMoV), and SARS-CoV-2 trends over time in a Selangor prison. These results support optimized WBE protocols for early outbreak detection in confined setting.

## 2. Materials and Methods

This study was conducted as part of a broader WBE research implemented at a prison facility in Selangor, Malaysia, as previously detailed by Rashid et al. (2025) [18]. While the earlier publication focused on comparing grab and composite sampling and reporting temporal SARS-CoV-2 trends, the present study centres on evaluating the analytical performance of two viral concentration methods, EMF and DC.

### 2.1. Study Site and Wastewater Sampling

This study took place at a prison facility in Selangor that housed approximately 800 inmates and staff. The facility comprises accommodation blocks, classrooms, communal sanitation facilities, and staff residential quarters, all serviced by a central wastewater network. Only methodological components relevant to the present comparison of concentration methods are described here. Additional details on the broader facility layout and routine operations are provided in Rashid et al. (2025) [18].

Wastewater sampling followed the established procedures but was adapted for this analytic comparison. From April to August 2023, influent wastewater was collected three times per week (Monday to Thursday) using a 24-h composite sampling approach. The ISCO 6712 autosamplers (Hoskin Scientific, Burlington, ON, Canada) collected 200 mL aliquots every four hours, yielding approximately 1.2 L per composite sample. Temperature was maintained at approximately ≤4 °C using dry ice throughout collection to limit RNA degradation.

All sampling activities were conducted in accordance with approved biosafety procedures [19]. Containers were pre-treated with RNase AWAY^®^ (Molecular BioProducts, San Diego, CA, USA), and personnel wore appropriate personal protective equipment (PPE). Upon arrival at the Institute for Medical Research (IMR), samples were ultraviolet (UV)-irradiated for 20 min inside a Class II biosafety cabinet to inactivate infectious material while preserving nucleic acid integrity. Several studies have shown that RT-PCR-detectable RNA, particularly for short, conserved targets such as the SARS-CoV-2 N gene, is not significantly impacted by UV treatment at relevant doses [20]. Samples were stored at −80 °C until processing.

### 2.2. Concentration and Extraction of SARS-CoV-2 RNA

The performance of two viral RNA concentration methods, EMF and DC, was evaluated.

#### 2.2.1. Electronegative Membrane Filtration and Extraction Method

For EMF, 39 mL of wastewater was mixed with 1 mL of 1 M MgCl_2_ (final concentration 25 mM) to enhance electrostatic adhesion of viral particles to the membrane. The mixture was filtered through a 0.45 µm pore-size, 47 mm-diameter HA membrane (Merck, Cork City, Ireland) under vacuum. After filtration, the sample tube was horizontally secured on a vortex adapter and shaken at maximum speed for 5 min to facilitate detachment of viral particles from the membrane surface. This was followed by centrifugation at 14,000× *g* for 1 min to remove large debris prior to RNA extraction. Viral RNA was extracted using the QIAGEN RNeasy PowerWater Kit (Qiagen, Hilden, Germany), which includes steps to remove inhibitors commonly found in wastewater. On-column DNase digestion was performed to eliminate carry-over DNA. Purified RNA was eluted in 100 µL of nuclease-free water and stored at −80 °C.

#### 2.2.2. Direct Capture Method

The direct capture (DC) workflow followed the procedure described by Rashid et al. (2025) [18]. Approximately 40 mL of wastewater was treated with 0.5 mL of protease and incubated at room temperature for 30 min. Following centrifugation at 5000× *g* for 10 min, the supernatant was combined with Binding Buffer 1, Binding Buffer 2, and isopropanol to a final volume of 50 mL. The mixture was applied to a PureYield^TM^ Midi Binding (Promega Corporation, Madison, WI, USA) column using a VacMan^®^ vacuum manifold (Promega Corporation, Madison, WI, USA), washed sequentially with column wash 1 (5 mL) and column wash 2 (20 mL), and eluted with 0.5 mL prewarmed nuclease-free water (60 °C). The eluate was then purified using the Maxwell^®^ RSC (Promega Corporation, Madison, WI, USA) system, yielding 80 µL of high-quality total nucleic acids.

### 2.3. RT-dPCR Detection of SARS-CoV-2

Quantification of SARS-CoV-2 RNA was performed using the GT-Digital SARS-CoV-2 Wastewater Surveillance Assay (GT Molecular, Fort Collins, CO, USA) on the QIAcuity dPCR 5-plex system (QIAGEN, Hilden, Germany). This workflow followed the methodology described by Rashid et al. (2025) [18] to compare viral RNA yields obtained from the two concentration methods.

Each 40 µL reaction contained the QIAGEN 4X One-Step Viral RT-PCR Master Mix, QIAGEN 100X Multiplex Reverse Transcription Mix, GT-Molecular 20X assay solution, nuclease-free water, and 20 µL of extracted nucleic acid. Gamma-irradiated SARS-CoV-2 RNA served as a positive control, while nuclease-free water served as the no-template control (NTC). PMMoV was quantified simultaneously to confirm the presence of human faecal material and to validate sample integrity.

Reaction mixtures were partitioned into 26,000 wells within nanoplates and amplified under the following cycling conditions: 50 °C for 30 min (reverse transcription), 95 °C for 2 min (enzyme activation), followed by 45 cycles of 95 °C for 10 s and 55 °C for 30 s. Fluorescence detection was automated, and absolute quantification was performed using QIAcuity Suite Software (v2.1.7.182). The analytical sensitivity of the assay was evaluated by determining the limit of blank (LOB) and Limit of Detection (LOD) [21]. The LOB was calculated from replicate NTC reactions as Mean_NTC_ + 1.645 × SD_NTC_. The LOD was subsequently determined as Mean_NTC_ + 3 × SD_low_, where SD_low_ represents the standard deviation of replicate measurements obtained from low-concentration positive samples near the detection limit (Refer to Supplementary Materials Table S1). This approach allows the lowest detectable concentration to be distinguished from background noise while minimizing false-positive results. Samples were considered positive when the concentration of either N1 or N2 exceeded the assay LOD. Concentrations were standardized to gene copies/L for statistical analysis.

### 2.4. Normalization Based on PMMoV

The overall faecal contribution to wastewater may vary daily as a result of fluctuations in flow rate, dilution from greywater inputs, and changes in contributing population size. Normalization using faecal indicators; PMMoV helps to account for this variability when interpreting SARS-CoV-2 RNA measurements. The viral RNA concentration was normalised to faecal content by calculating the viral copies per faecal content equivalent value for each sample and site. Normalization parameters were calculated as follows:

*CPMMoV Normalized* = *C*/*P*

*CPMMoV Normalized* = Concentration of SARS-CoV-2 RNA in copies/L wastewater, normalized for PMMoV concentration.

*C* = Concentration of SARS-CoV-2 RNA in copies/L wastewater

*P* = Concentration of PMMoV in copies/L wastewater

### 2.5. Statistical Analysis

Statistical analyses were conducted to compare the performance of EMF and DC concentration methods and to evaluate differences between the SARS-CoV-2 N1 and N2 targets. SARS-CoV-2 RNA concentrations were matched by sampling date. Zero values were replaced with half of the assay limit of detection (LOD/2) prior to log_10_ transformation to accommodate non-detects [22].

Paired Wilcoxon signed-rank tests were used to assess differences in SARS-CoV-2 RNA concentrations between EMF and DC methods separately for N1 and N2. To evaluate whether N1 concentrations were higher than N2, paired one-sided Wilcoxon signed-rank tests were performed within each concentration method (DC and EMF). Normality of paired differences was assessed using the Shapiro–Wilk test to inform test selection.

Agreement between measurements was evaluated using Pearson and Spearman correlation coefficients to assess linear and monotonic associations, respectively, and Bland–Altman analysis was applied on log_10_-transformed concentrations to estimate mean bias and 95% limits of agreement. Comparisons between raw and PMMoV-normalized SARS-CoV-2 RNA concentrations for the DC method were conducted using the same statistical approaches.

All statistical analyses were performed using R (version 4.5.2). Unless otherwise specified, statistical tests were two-sided, with statistical significance defined as *p* < 0.05.

## 3. Results

### 3.1. SARS-CoV-2 RNA Detection by Concentration Method

The detection and quantification of SARS-CoV-2 RNA in wastewater samples varied depending on the concentration method and the target gene. Using the EMF method, the median SARS-CoV-2 RNA concentrations were 0.70 × 10^3^ gene copies L^−1^ for the N1 gene and 0.48 × 10^3^ gene copies L^−1^ for the N2 gene. The interquartile ranges (IQR) were 3.66 × 10^3^ gene copies L^−1^ (N1) and 2.13 × 10^3^ gene copies L^−1^ (N2), with observed ranges extending up to 38.26 × 10^3^ gene copies L^−1^ and 16.96 × 10^3^ gene copies L^−1^, respectively. The EMF method detected SARS-CoV-2 RNA in 88% (*n* = 44) of samples for the N1 target and 76% (*n* = 38) for the N2 target, indicating moderately high detection frequencies. In comparison, the Direct Capture (DC) method produced substantially higher concentrations for both targets. The median RNA concentrations were 11.09 × 10^3^ gene copies L^−1^ for N1 and 3.43 × 10^3^ gene copies L^−1^ for N2, with IQR values of 35.96 × 10^3^ and 10.02 × 10^3^ gene copies L^−1^, respectively. The observed concentration ranges extended up to 302.76 × 10^3^ gene copies L^−1^ for N1 and 239.36 × 10^3^ gene copies L^−1^ for N2. The proportion of positive detections was also higher for the DC method, with 94% (*n* = 47) of samples testing positive for N1 and 84% (*n* = 42) for N2.

As summarised in Table 1, the EMF method yielded lower median concentrations and lower detection frequencies for both N1 and N2 compared to the DC method. In contrast, the DC method consistently produced higher viral RNA concentrations, broader concentration ranges, and a greater proportion of positive detections. Across both methods, the one-sided Wilcoxon signed-rank test indicated that N1 concentrations were significantly higher than N2 (*p* < 0.001 for both comparisons).

### 3.2. Comparison of SARS-CoV-2 Detection Efficiency Between Direct Capture and Electronegative Membrane Filtration

**Figure 1:**
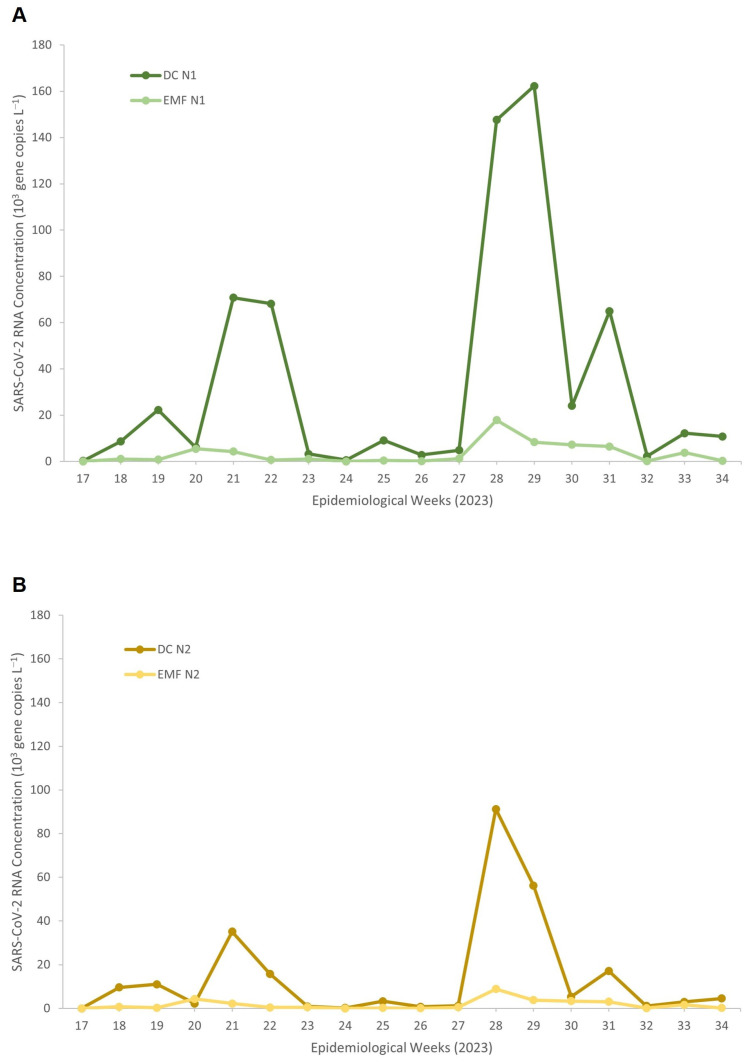
SARS-CoV-2 RNA concentrations measured by the Direct Capture and Electronegative Membrane method for (**A**) N1 and (**B**) N2 targets across epidemiological weeks in 2023.

Figure 1 illustrates the temporal trends in SARS-CoV-2 RNA concentrations detected using DC and EMF methods across epidemiological weeks. For both N1 (Figure 1A) and N2 (Figure 1B) gene targets, DC consistently yielded higher viral RNA concentrations compared to EMF. DC detected distinct temporal peaks, including marked increases during epidemiological weeks (EW) 21–22 (May), EW 28–29 (July), and EW 31 (August). The most prominent increase occurred around EW 28–29 (July), where these peaks were consistently detected across both gene targets, demonstrating temporal concordance and reinforcing the robustness of the DC-based signal likely reflecting periods of increased viral circulation within the facility. In contrast, EMF showed substantially lower concentrations with minimal variation over the 18-week surveillance period, indicating reduced analytical sensitivity and limited ability to detect changes in viral load over time. These findings demonstrate that the DC method yields superior viral recovery and more reliable trend detection than EMF for wastewater-based surveillance (WBS) in this prison setting.

Statistical comparison using daily paired measurements (Table 2) showed that DC consistently produced significantly higher SARS-CoV-2 RNA concentrations than EMF for both target genes, N1 (*p* = 2.31 × 10^−7^) and N2 (*p* = 9.40 × 10^−5^), based on Wilcoxon signed-rank test. Correlations analyses between DC and EMF revealed only moderate associations for N1 (Pearson *r* = 0.472; Spearman ρ = 0.659) and weak-to-moderate associations for N2 (Pearson *r* = 0.392; Spearman ρ = 0.534), indicating inconsistent agreement between methods. Bland–Altman analysis further confirmed substantial negative biases for both N1 (−1.15 log_10_) and N2 (−0.87 log_10_), with wide limits of agreement, demonstrating that EMF consistently underestimated SARS-CoV-2 RNA concentrations relative to DC and that the two concentration methods are not interchangeable.

### 3.3. Effect of PMMoV Normalization on SARS-CoV-2 RNA Concentrations

**Figure 2:**
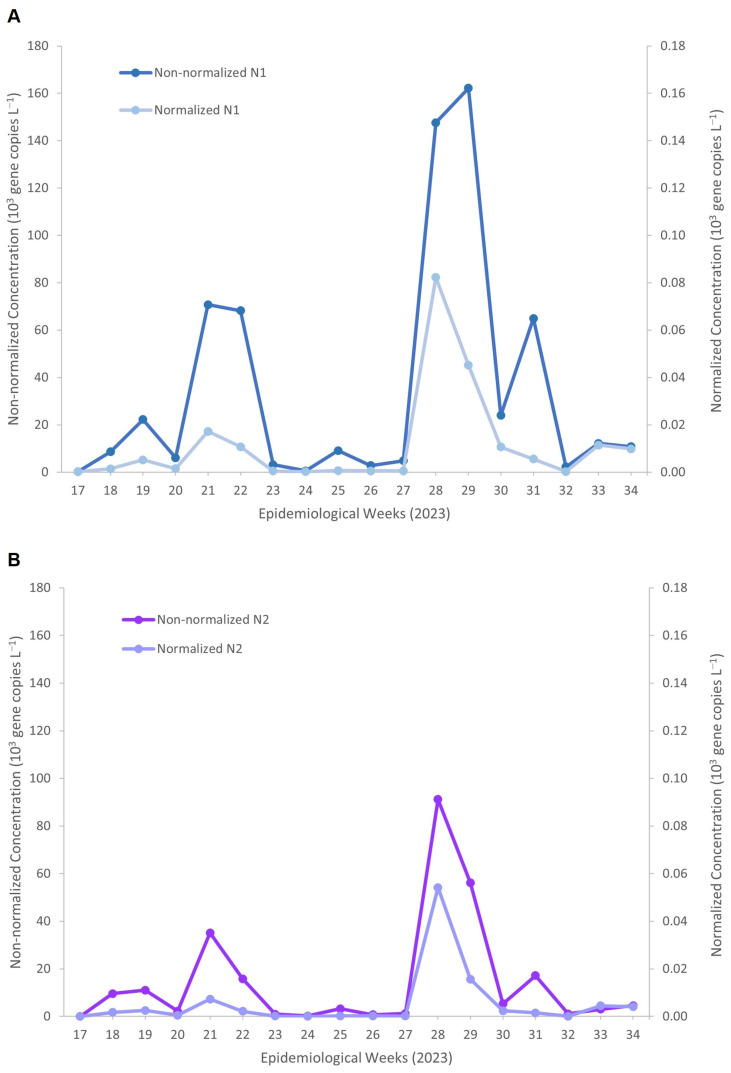
Temporal profiles of non-normalized and PMMoV-normalized SARS-CoV-2 RNA concentrations measured by the Direct Capture method for (**A**) N1 and (**B**) N2 targets across epidemiological weeks in 2023. Non-normalized concentrations are shown on the left *y*-axis, and PMMoV-normalized concentrations on the right *y*-axis.

Figure 2 illustrates the impact of PMMoV normalization on SARS-CoV-2 RNA measurements obtained using the DC method. For both N1 (Figure 2A) and N2 (Figure 2B), normalizing SARS-CoV-2 RNA concentrations to PMMoV, a faecal-strength biomarker commonly used to account for population and wastewater dilution variability, resulted in noticeably lower absolute concentrations than the non-normalized concentrations. Despite this reduction in magnitude, the normalized trends closely mirrored the temporal dynamics observed in the non-normalized N1 concentrations, reaching a maximum of approximately 162 × 10^3^ gene copies L^−1^. This included distinct peaks during epidemiological weeks 21–22 (68–77 × 10^3^ gene copies L^−1^) and weeks 28–29 (147–162 × 10^3^ gene copies L^−1^).

After PMMoV normalization, peak concentrations were reduced to approximately 0.05 × 10^3^ gene copies L^−1^, corresponding to an overall reduction of about 3 log_10_ units while preserving the timing and relative magnitude of temporal peaks. Similarly, for the N2 target (Figure 2B), non-normalized concentrations peaked at approximately 91 × 10^3^ copies L^−1^ during week 28, whereas PMMoV-normalized concentrations reached a maximum of approximately 0.05 × 10^3^ copies L^−1^, reflecting a comparable ~3 log_10_ reduction.

Paired testing (Table 3) showed that PMMoV normalization significantly reduced SARS-CoV-2 RNA concentrations for both N1 (*p* = 2.48 × 10^−9^) and N2 (*p* = 1.71 × 10^−8^). Despite the reduction in magnitude, normalized and non-normalized concentrations remained strongly correlated (N1: Pearson *r* = 0.662; Spearman ρ = 0.876; N2: Pearson r = 0.475; Spearman ρ = 0.786), indicating the preservation of temporal trends. Bland–Altman analysis showed consistent negative biases for N1 (−3.46 log_10_) and N2 (−3.11 log_10_), with all limits of agreement below zero, demonstrating that normalization acts as a downward scaling factor without altering the relative ranking of the sample.

## 4. Discussion

This study presents the performance of two commonly used viral RNA concentration methods, DC and EMF, for detecting SARS-CoV-2 RNA in wastewater collected from a prison setting. Electronegative membrane filtration (EMF) concentrates SARS-CoV-2 RNA by promoting electrostatic adsorption of viral particles onto a negatively charged membrane using Mg^2+^ cations supplied by magnesium chloride (MgCl_2_), followed by separate RNA extraction. Direct capture (DC) employs chemical binding of viral RNA within an integrated concentration–extraction workflow, reducing handling steps and improving consistency under variable wastewater conditions. The findings demonstrate clear methodological differences: DC consistently outperforms EMF across multiple analytical indicators, including viral recovery, detection frequency, and temporal trend capture in viral load across both target genes.

Across the study period, SARS-CoV-2 RNA concentrations measured using the DC method were significantly higher than those obtained with EMF for both N1 and N2 gene targets. Temporal trend analysis showed that DC detected distinct increases in viral concentrations in May, July and August, reflecting changes in viral circulation within the facility. The wide IQR and range observed for the DC method reflect variability in viral load across samples but also highlight the method’s capacity to capture low and high viral RNA concentrations effectively. These findings indicate that DC coupled with the N1 target provides the most sensitive and robust approach for SARS-CoV-2 RNA detection in wastewater from confined settings. In contrast, EMF produced lower, more stable signals with limited week-to-week variation, suggesting reduced analytical sensitivity. Similar challenges with EMF have been reported in previous studies, in which electronegative membranes demonstrated lower recovery efficiency and greater susceptibility to inhibitors in raw wastewater [23,24,25]. These findings indicate that DC is more suitable for detecting subtle fluctuations in SARS-CoV-2 shedding, particularly in low-abundance environments.

Statistical analyses further supported these observations. Paired testing demonstrated that DC consistently yielded significantly higher viral RNA concentrations than EMF, and agreement analyses indicated only moderate to weak correlations between the two methods. Bland–Altman analysis confirmed that EMF systematically underestimated SARS-CoV-2 RNA concentrations, a trend also highlighted in comparative evaluations of concentration techniques conducted in other studies [17,26]. Together, these results confirm that EMF and DC are not analytically interchangeable and that the selected concentration method can substantially influence the interpretation of wastewater SARS-CoV-2 signals.

Differences among gene targets carry substantial analytical implications for SARS-CoV-2 surveillance via wastewater. The superior performance of the N1 target, as observed in this study and prior research, indicates that assay-specific properties rather than authentic variations in viral load predominantly account for discrepancies between markers, likely due to differences in amplicon stability, amplicon efficiency, and mismatch tolerance [18,27,28]. These characteristics likely bolster N1’s reliability in complex wastewater settings, where RNA degradation and inhibitory compounds prevail. However, these findings should be interpreted with caution, as the observed differences between N1 and N2 are likely influenced by assay-specific performance characteristics rather than definitive differences in viral abundance. Therefore, conclusions regarding target superiority should remain context-dependent and supported by multi-target validation where possible. However, it is important to note that other studies have reported conflicting results, with some finding N2 to be more sensitive or detecting higher viral quantities than N1, underscoring the ongoing debate and the need for standardized protocols in SARS-CoV-2 wastewater surveillance [29,30]. Sherchan et al. (2020) [31], who also noted a discrepancy between N1 and N2 assays, have discussed possible factors responsible for this inconsistency, namely, primer and probe sequences, assay sensitivity, low levels of SARS-CoV-2 RNA in wastewater, and subsampling error. In addition, stochastic variation in viral shedding within wastewater systems may influence assay detection patterns, particularly in relatively small catchment areas where viral signals may be dominated by a limited number of infected individuals.

In a related study conducted at the same prison site (Rashid et al., 2025) [18], genomic sequencing of wastewater samples identified multiple circulating Omicron sublineages and mutations across the SARS-CoV-2 genome, including within the nucleocapsid (N) gene. SNV frequency data generated using the Freyja pipeline showed temporal variability in mutations such as C28311T (N:P13L), located within the N1 target region (Figure 6 in [18]). To assess whether such mutations contributed to differences in assay performance, we re-examined SNV frequency data with specific focus on primer–probe binding regions. While temporal variability was observed within the N1 region, no consistent or high-frequency SNVs were detected within the N2 primer–probe binding regions during the study period.

These findings suggest that mutations may have contributed to variability in N1 detection but are unlikely to explain the comparatively lower sensitivity observed for N2. Instead, differences in assay design, amplification efficiency, and target stability are likely to play a more substantial role. Nevertheless, the presence of multiple co-circulating variants within a relatively small catchment population may introduce stochastic variability in wastewater signals, particularly in confined settings such as prisons where supershedding events may occur. Further work incorporating detailed primer–probe mapping and evaluation of mismatch effects on amplification efficiency is warranted to better elucidate the impact of mutations on assay performance.

In another study, Endo et al. (2024) [32], reported that SARS-CoV-2 RNA concentrations estimated using the CDC N1 assay diverged significantly from those derived from the N2 assay, this was not due to a decline in actual viral load, but rather a failure of the qPCR analysis to accurately quantify specific viral mutation in the standard N1 assay parameters. This phenomenon has been cited by other researchers as a cautionary example of how mutations can compromise quantification accuracy. Unlike quantitative PCR (qPCR), dPCR is an endpoint assay that counts positive partitions rather than relying on amplification kinetics [32]. Consequently, it is less susceptible to the fluorescence signal attenuation caused by probe mismatches.

To mitigate potential analytical “blind spots” caused by region-specific mutations, the simultaneous analysis of multiple genomic targets is therefore recommended rather than reliance on a single marker. Routine monitoring of the relative performance or ratio between targets such as N1 and N2 provides an additional quality-control measure [33,34,35], as substantial deviations from the expected ratio of approximately one may indicate mutation-driven assay bias. Nevertheless, the consistently higher sensitivity observed for N1 in this study suggests that well-optimised N1 assays alone can yield representative SARS-CoV-2 wastewater signals, particularly when coupled with RT-dPCR, which enhances robustness against amplification inefficiencies. While dual-target detection offers analytical redundancy, these findings support the continued use of N1 as a primary target for wastewater-based SARS-CoV-2 epidemiology.

The strong correlations observed between non-normalized and PMMoV-normalized concentrations suggest that normalization can account for variability in wastewater dilution, faecal load, and contributing population size while largely preserving temporal patterns of SARS-CoV-2 RNA. Therefore, both normalized and non-normalized SARS-CoV-2 RNA concentrations were evaluated to assess their consistency and epidemiological relevance. Although normalization reduced absolute concentrations across both gene targets, peak timing and trend direction remained consistent. This aligns with prior findings indicating that PMMoV normalization improves data comparability without distorting epidemiological trends [36,37,38]. However, the interpretation of PMMoV normalization in small catchments requires caution. PMMoV is introduced into wastewater primarily through the consumption of pepper-containing foods, and therefore its concentration may be influenced by dietary patterns [39]. In smaller populations such as institutional facilities, day-to-day dietary changes may contribute to fluctuations in PMMoV concentrations [37]. In the present study, meals within the prison facility are centrally prepared and distributed according to a standardized schedule, meaning inmates receive similar meals on a given day. This structured dietary provision likely reduces large day-to-day variations in PMMoV across the population compared with communities where individuals consume highly variable diets. Nevertheless, dietary-driven variability cannot be completely excluded and should be considered when interpreting PMMoV-normalized viral concentrations in small institutional catchments. Furthermore, several studies have reported that PMMoV normalization does not consistently improve correlations with epidemiological indicators and may introduce additional variability depending on site-specific conditions. As such, normalized data should be interpreted alongside raw concentrations, particularly in small or relatively stable catchment populations. To address this limitation, some studies have incorporated additional normalization biomarkers, such as crAssphage, which may provide complementary indicators of human fecal input in wastewater surveillance [40,41].

In prisons where mixed grey- and blackwater streams, fluctuating occupancy, abrupt population shifts, inmate movements, and visitor and staff activity are common [11] these findings highlight the utility of PMMoV normalization for improving interpretability and strengthening the reliability of wastewater-based early outbreak detection, particularly with the superior PMMoV recovery achieved via the DC method. PMMoV normalization may not solely correct for dilution but may also capture short-term changes in the contributing population. Similarly, a study in Alberta found that using unnormalised concentrations often provided value equivalent to PMMoV normalisation without the added cost [42].

Importantly, wastewater surveillance in such settings is typically interpreted as a trend-based early warning system rather than relying on a strict detection threshold. Detection of SARS-CoV-2 RNA above zero copies does not necessarily indicate active transmission, as low-level signals may reflect residual shedding or sporadic viral contributions. Instead, sustained increases in viral RNA concentrations or repeated detections across consecutive sampling events are generally considered more informative indicators of potential transmission within the monitored population. This enhanced sensitivity may facilitate earlier identification of meaningful transmission signals, supporting timely public health action in confined settings. In a prison setting, such signals could prompt targeted public health responses, including enhanced clinical testing of inmates and staff, reinforcement of infection prevention measures, isolation of symptomatic individuals, or temporary cohorting strategies to reduce transmission risk. Within this framework, PMMoV normalization may help improve interpretation of viral trends by accounting for variations in fecal load and wastewater dilution, thereby supporting more reliable detection of meaningful epidemiological changes.

Collectively, these findings highlight the operational advantages of the DC method for WBE. Its higher viral recovery, greater sensitivity to temporal fluctuations, and stronger detection performance make DC better suited for early warning systems in confined or high-risk environments, where clinical testing may be constrained, and outbreaks can escalate rapidly. Wastewater surveillance has been shown to correlate with community infection trends and, in some settings, to provide early warning of rising case incidence before clinical reporting [43,44]. Ensuring the use of highly sensitive concentration methods is therefore essential for the timely and reliable detection of viral circulation in such environments.

### 4.1. Strengths and Limitations

This study is among the few evaluations that directly compare EMF and DC in a field-based, confined institutional setting, providing practical insights relevant to WBS systems. Unlike controlled laboratory comparisons, this study examines performance under authentic wastewater conditions, strengthening ecological validity. Sampling carried over 5 months enabled a robust evaluation of temporal sensitivity, allowing assessment of each method’s ability to detect fluctuations in SARS-CoV-2 viral load over time. Quantification using dPCR achieved high analytical precision, minimizing stochastic measurement error and improving the reliability of concentration comparisons across methods and gene targets. The inclusion of PMMoV normalization provides additional interpretive value by demonstrating how faecal-strength adjustment affects the preservation of SARS-CoV-2 trend patterns and comparability.

However, several limitations should be acknowledged. This study was conducted in a single prison facility, and results may not be generalizable to wastewater systems with different hydraulic properties, population behaviours, or levels of organic and chemical load. Only influent wastewater was assessed, limiting spatial resolution; upstream sampling within the sewer network could provide greater insight into localized shedding patterns. The comparison was restricted to two concentration methods, although other widely used approaches, such as PEG precipitation, ultrafiltration, and ultracentrifugation, may have performed differently. The inherent variability of wastewater composition, including fluctuations in solid content and the presence of inhibitory substances, may have influenced viral recovery despite the use of standardized protocols.

### 4.2. Recommendations

Future studies should investigate the underlying factors contributing to differences in viral recovery between the DC and EMF methods, including the influence of wastewater composition, inhibitors, and solids content, which were not directly assessed in this study. Additional work is needed to quantify method-specific recovery efficiencies through controlled spiking experiments, enabling more accurate adjustment and interpretation of viral concentrations in real-world surveillance. Future research should also assess the operational feasibility, cost-effectiveness, and scalability of the DC method in routine surveillance workflows, particularly in facilities with limited resources or technical capacity. Finally, evaluating the performance of these concentration methods for other pathogens of public health importance, beyond SARS-CoV-2, would help determine whether the observed advantages of DC extend to broader wastewater-based infectious disease monitoring.

## 5. Conclusions

This study highlights the critical influence of concentration method selection on the accuracy and responsiveness of wastewater-based SARS-CoV-2 surveillance. By demonstrating that DC substantially enhances viral recovery, detection frequency, and temporal signal clarity compared to EMF, our findings reinforce the need for methodological optimization in wastewater monitoring frameworks, particularly in high-risk institutional environments. The significantly better performance of the N1 gene further underscores the importance of target selection when interpreting viral dynamics in complex wastewater matrices. Although PMMoV normalization reduced absolute SARS-CoV-2 RNA concentrations, it preserved trend fidelity, supporting its value in stabilizing data across variable sampling conditions. Together, these results strengthen the evidence base for refining analytical workflows in wastewater surveillance and provide actionable insights to improve early-warning capability in confined settings, where timely detection is essential. Continued methodological evaluation will be important as WBE evolves to support broader infectious disease monitoring and public health decision-making.

## Figures and Tables

**Table 1:** Descriptive statistics of SARS-CoV-2 RNA concentrations measured for EMF and DC methods.

Concentration Method	Target Gene	RNA Concentration (10^3^ Gene Copies L^−1^)		Range	Positive Samples, *n* (%)	Normality of log_10_ Difference (Shapiro–Wilk *p*)	*p*-Value
		Median	IQR				
EMF	N1	0.70	3.66	ND–38.26	88 (44)	8.56 × 10^−10^	1.14 × 10^−6^
	N2	0.48	2.13	ND–16.96	76 (38)		
DC	N1	11.09	35.96	ND–302.76	94 (47)	1.53 × 10^−9^	8.75 × 10^−9^
	N2	3.43	10.02	ND–239.36	84 (42)		

EMF: Electronegative membrane method, DC: Direct Capture method. The IQR represents the range within which the middle 50% of the data lie.

**Table 2:** Comparison of DC and EMF for raw SARS-CoV-2 concentrations.

Target	Paired Test	*p*-Value	Pearson *r*	Spearman ρ	Mean Bias	95% LoA (Lower, Upper)
N1	Wilcoxon signed-rank	2.25 × 10^−7^	0.472	0.659	−1.15	−3.85 to 1.24
N2	Wilcoxon signed-rank	9.19 × 10^−5^	0.392	0.534	−0.87	−4.27 to 2.53

LoA: Limit of agreement.

**Table 3:** Comparison of raw and PMMoV-normalized SARS-CoV-2 RNA concentrations for the DC method.

Target	Paired Test	*p*-Value	Pearson *r*	Spearman ρ	Mean Bias	95% LoA (Lower, Upper)
N1	Wilcoxon signed-rank	2.40 × 10^−9^	0.662	0.876	−3.46	−5.37 to 1.54
N2	Wilcoxon signed-rank	1.65 × 10^−8^	0.475	0.786	−3.11	−5.90 to −0.31

LoA: Limit of agreement.

## Data Availability

The original contributions presented in this study are included in the article/Supplementary Material. Further inquiries can be directed to the corresponding author.

## Supplementary Materials

The following supporting information can be downloaded at: https://www.mdpi.com/article/10.3390/v18050563/s1, Table S1: Estimated Limit of blank (LOB) and Limit of detection (LOD) for N1 and N2. Unit of measurement is in copies/µl.
