# Supplementary material for: Comparative Evaluation of SARS-CoV-2 RNA Concentration and Normalization Strategies in Prison Wastewater: Implications for Viral Dynamics in Confined Environments
Source: Viruses. 2026 May 15;18(5):563. doi: 10.3390/v18050563 (PMC13211580; doi:10.3390/v18050563)
Supplement: Supplementary file 1 [file viruses-18-00563-s001.zip › viruses-4198965-supplementary.pdf]

Supplementary File

Table S1: Estimated Limit of blank (LOB) and Limit of detection (LOD) for N1 and N2. Unit of measurement is in copies/µl

Target	$Mean_{NTC}$	SD_{NTC}	SD_{low}	LOB	LOD
**N1**	0.0000	0.0000	0.0017	0.0000	0.0051
**N2**	0.0000	0.0000	0.0011	0.0000	0.0033

Limit of Detection (LOD)

Target: N1

LOD $= Mean_{NTC} + (3*SD_{NTC})$

$= 0.0000 + (3*0.0017)$

$= 0.0051$

Target: N2

LOD $= Mean_{NTC} + (3*SD_{NTC})$

$= 0.0000 + (3*0.0011)$

$= 0.0033$
